# Breast Cancer 1 (BrCa1) May Be behind Decreased Lipogenesis in Adipose Tissue from Obese Subjects

**DOI:** 10.1371/journal.pone.0033233

**Published:** 2012-05-30

**Authors:** Francisco J. Ortega, José M. Moreno-Navarrete, Dolores Mayas, Eva García-Santos, María Gómez-Serrano, José I. Rodriguez-Hermosa, Bartomeu Ruiz, Wifredo Ricart, Francisco J. Tinahones, Gema Frühbeck, Belen Peral, José M. Fernández-Real

**Affiliations:** 1 Service of Diabetes, Endocrinology and Nutrition, Institut d'Investigació Biomèdica de Girona, CIBER de la Fisiopatología de la Obesidad y Nutrición, and Instituto de Salud Carlos III, Girona, Spain; 2 Service of Endocrinology and Nutrition, Hospital Clínico Universitario Virgen de Victoria de Malaga, CIBER de la Fisiopatología de la Obesidad y Nutrición, and Instituto de Salud Carlos III, Málaga, Spain; 3 Department of Endocrinology, Physiopathology and Nervous System, Instituto de Investigaciones Biomédicas ‘Alberto Sols’, Consejo Superior de Investigaciones Científicas and Universidad Autónoma de Madrid, Madrid, Spain; 4 Department of Surgery, Institut d'Investigació Biomèdica de Girona, Girona, Spain; 5 Department of Endocrinology, Clínica Universidad de Navarra, CIBER de la Fisiopatología de la Obesidad y Nutrición, Instituto de Salud Carlos III, Navarra, Spain; University of Cordoba, Spain

## Abstract

**Context:**

Expression and activity of the main lipogenic enzymes is paradoxically decreased in obesity, but the mechanisms behind these findings are poorly known. Breast Cancer 1 (BrCa1) interacts with acetyl-CoA carboxylase (ACC) reducing the rate of fatty acid biosynthesis. In this study, we aimed to evaluate BrCa1 in human adipose tissue according to obesity and insulin resistance, and *in vitro* cultured adipocytes.

**Research Design and Methods:**

BrCa1 gene expression, total and phosphorylated (P-) BrCa1, and ACC were analyzed in adipose tissue samples obtained from a total sample of 133 subjects. BrCa1 expression was also evaluated during *in vitro* differentiation of human adipocytes and 3T3-L1 cells.

**Results:**

*BrCa1* gene expression was significantly up-regulated in both omental (OM; 1.36-fold, p = 0.002) and subcutaneous (SC; 1.49-fold, p = 0.001) adipose tissue from obese subjects. In parallel with increased *BrCa1* mRNA, P-ACC was also up-regulated in SC (p = 0.007) as well as in OM (p = 0.010) fat from obese subjects. Consistent with its role limiting fatty acid biosynthesis, both *BrCa1* mRNA (3.5-fold, p<0.0001) and protein (1.2-fold, p = 0.001) were increased in pre-adipocytes, and decreased during *in vitro* adipogenesis, while P-ACC decreased during differentiation of human adipocytes (p = 0.005) allowing lipid biosynthesis. Interestingly, *BrCa1* gene expression in mature adipocytes was restored by inflammatory stimuli (macrophage conditioned medium), whereas lipogenic genes significantly decreased.

**Conclusions:**

The specular findings of BrCa1 and lipogenic enzymes in adipose tissue and adipocytes reported here suggest that BrCa1 might help to control fatty acid biosynthesis in adipocytes and adipose tissue from obese subjects.

## Introduction

The complex process of differentiation by which new fat cells are developed from pre-adipocytes is known as adipogenesis. During this process, the most dramatic changes are observed in relation to structural changes that allow the biosynthesis of lipids (or lipogenesis). Fatty acid synthase (FASN; EC 2.3.1.85) and acetyl-CoA carboxylase (ACC; EC 6.4.1.2) are examples of master enzymes in lipogenesis [1], [2]. The later, ACC, catalyses the formation of malonyl-CoA, an essential substrate for FASN and the chain elongation systems [2], [3]. ACC is present in the cytosol of all tissues and is especially enriched in adipose tissue and liver. The acute control of ACC activity is the product of integrated changes such as the phosphorylation of multiple serine residues and interactions with other proteins [see [3] for references].

The expression of lipogenic enzymes is decreased in overweight and obese subjects [4], [5], [6], [7], [8]. However, the hyperplasic component of adipose tissue is currently well recognized and refers both to the recruitment and proliferation of adipocyte precursor cells (also named pre-adipocytes) [9], [10], [11] followed by adipogenesis [12], [13]. Since the development of obesity involves an extensive adipose tissue remodeling which is dependent on the coordinated interplay between adipocyte hypertrophy (increase in cell size assessed during adipogenesis) and adipocyte hyperplasia (increase in cell number) [14], [15], the before mentioned findings seem to be contrary to what might be expected.

Otherwise, it is well known that chronic subclinical inflammation is intrinsic to the metabolic syndrome (the clustering of central obesity and alterations of glucose and lipid metabolism). Insulin resistance is central to the pathophysiology of these alterations, which runs together with the accumulation of fat and the presence of specific components that might be of importance in the development of type 2 diabetes (T2D) [16]. In this respect, the transcription factor sterol regulatory element binding protein (SREBP)-1c transduces the insulin signal in insulin sensitive tissues such as adipose tissue, and its involvement in lipogenic genes which require for their expression both insulin and glucose (namely FAS and ACC) is currently well recognized [17].

Breast Cancer 1 (BrCa1) is a ∼220-KDa protein involved in multiple cellular functions such as DNA repair, cell cycle checkpoint control, transcription, and ubiquitination [18], [19]. BrCa1 mRNA is primarily expressed in a large variety of epithelia in tissues derived from the ectoderm, endoderm, and mesoderm [20]. Widespread tissue- and cell-specific expression of the *BrCa1* transcript in mammalians has been reported [21], [22]. Although BrCa1 has been shown to have tumor-suppressive properties in breast and ovarian cells, its broad distribution and its crucial role in the development of different germ layers during embryogenesis [23], [24], [25] suggest a more generalized role that includes both differentiation [21], [22] and cell proliferation [23], [24]. This data suggest an important developmental role for BrCa1 in the regulation of proliferation and differentiation of many cell lines, but nowadays little is known about BrCa1 function and the regulation of its expression in human fat depots, pre-adipocytes and mature adipocytes.

Recent studies have provided structural evidence for direct interactions between the BrCa1 C-Terminal (BRCT) domains and a phospho-peptide from the cytosolic enzyme ACC [26]. It has been suggested that BrCa1 helps to maintain fatty acid biosynthesis and lipogenesis under control in normal cells, whereas mutations in the BRCT domains of BrCa1 can abolish the regulation of ACC and lead to elevated lipogenesis, which is an important requirement for cancer cell growth [27], [28]. Otherwise, when BrCa1 interacts with ACC preventing ACC dephosphorylation, the rate of fatty acid biosynthesis seems to be significantly reduced [29], [30], [31]. Therefore, if BrCa1 helps to control fatty acid biosynthesis in normal cells, it is important to understand this regulation in the tissue where the most dramatic changes in lipid biosynthesis take place (i.e. adipose tissue) in association with the presence of obesity and alterations of glucose and lipid metabolism. Of note, insulin resistance was recently associated with an expanded population of small adipocytes, suggesting impairment in adipose cell differentiation in obese subjects with impaired glucose tolerance [32]. On the other hand, obese individuals often have enlarged adipocytes with a reduced buffering capacity for lipid storage leading to ectopic fat deposition and insulin resistance [33].

Interestingly, BrCa1 was found to be up-regulated in adipose tissue from obese subjects independently of T2D. To gain further insight into the meaning of this finding, we also analyzed *BrCa1* mRNA during *in vitro* differentiation of human pre-adipocytes, in mature adipocytes treated with inflammatory stimuli (macrophage conditioned medium), and both BrCa1 and phosphorylated-BrCa1 protein in 3T3-L1, in parallel to phosphorylation of ACC.

## Results

### *BrCa1* levels in human fat samples

**Figure 1:**
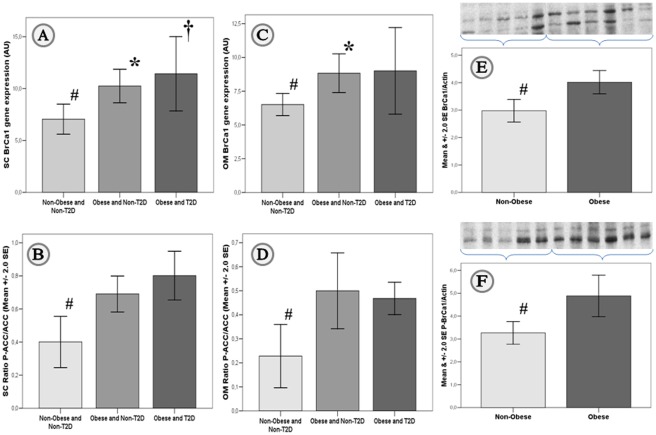
*BrCa1* levels in human fat samples. *Real Time-PCR:* Mean and 95% confidence interval for the mean of gene expression levels for *BrCa1* in subcutaneous (SC; *Fig. 1a*) and omental (OM; *Fig. 1c*) adipose tissue from non-obese (BMI<30 kg/m^2^) and obese (BMI≥30 kg/m^2^) subjects with and without T2D. *ELISA:* Mean ±2.0 SE for phosphorylated ACC normalized *versus* total ACC (ratio P-ACC/ACC) in SC (*Fig. 1b*; n = 33) and OM (*Fig. 2d*; n = 21) adipose tissue. *Western-blot:* Mean ±2.0 SE of total (BrCa1, *Fig. 2e*) and phosphorylated (P-BrCa1; *Fig. 2f*) BrCa1 protein normalized *versus* β-actin in OM fat. ***†*** and * p<0.05 for comparisons between obese with and without T2D, and the control group (non-obese and non-T2D individuals), respectively. ***^#^*** p<0.05 for comparisons between non-obese and obese subjects.

The anthropometric and metabolic characteristics of the study subjects are shown in ***Table 1***. Obese subjects with type 2 diabetes (T2D) were identified from obese outpatient clinics on the basis of a stable metabolic control in the previous 6 months, as defined by stable HbA1c and fasting glucose values. *BrCa1* gene expression levels in both subcutaneous (1.46-fold, p = 0.005; ***Fig. 1a***) and omental (1.36-fold, p = 0.002; ***Fig. 1c***) fat depots, as well as total (***Fig. 1e***) and phosphorylated (***Fig. 1f***) BrCa1 protein (1.35-fold, p = 0.007, and 1.50-fold, p = 0.016, respectively), were significantly increased in obese when compared to non-obese subjects. Concomitantly, *SREBP-1c*, *FASN* and *ACC* gene expression levels were down-regulated in adipose tissue from obese subjects (***Table 1***). *BrCa1* expression was significantly increased by 1.34-fold (p = 0.001) in SC versus OM adipose tissue (n = 51-paired samples; ***Figure S1a***). Gene expression measures for *BrCa1* in OM and SC fat were significantly associated (r = 0.37, p = 0.004).

**Table 1:** Anthropometrical and biochemical characteristics of study subjects.

*Whole cohort*	*A. Non-Obese*	*B. Obese*	*C. Obese and T2D*	*P (ANOVA)*	*P (t-Student)*
*n (SC)*	18	59	19		
*Sex (% women)*	67	61	68		
*Age (years)*	46±10	43±10	47±12	0.251	0.337
*BMI (kg/m^2^)*	**26.5±2.3**	**43.6±7.0**	**45.4±7.8**	***<0.0001***	***<0.0001***
*Fasting glucose (mg/dL)*	**85.9±10.6**	**95.2±14.1**	**116.5±17.0**	***<0.0001***	***<0.0001***
*Fasting insulin (µUI/mL)* †	**6.7±1.4**	**13.5±7.9**	**22.1±19.3**	***0.032***	0.112
*HOMA-IR* †	**1.42±0.23**	**3.46±2.46**	**6.45±5.64**	***0.024***	0.142
*HbA_1c_ (%)*	**4.9±0.6**	**4.9±0.5**	**5.1±0.4**	***<0.0001***	0.622
*SC BrCa1 (mRNA)*	**7.0±3.1**	**10.2±6.2**	**11.4±7.4**	0.072	***0.001***
*SC FASN (mRNA)*	**11.4±14.9**	**3.6±5.3**	**6.1±4.8**	***0.026***	0.175
*SC ACC (mRNA)*	**4.3±4.4**	**2.5±2.3**	**1.5±1.1**	***0.031***	0.088
*SC SREBP-1c (mRNA)*	**1.9±2.1**	**1.1±0.6**	**1.0±0.4**	***0.033***	0.084
*n (OM)*	38	38	12		
*Sex (% women)*	65	53	58		
*Age (years)*	47±14	43±11	47±10	0.301	0.249
*BMI (kg/m^2^)*	**23.19±2.53**	**44.18±7.73**	**45.17±7.79**	***<0.0001***	***<0.0001***
*Fasting glucose (mg/dL)*	**79.74±8.08**	**94.71±12.94**	**114.58±20.82**	***<0.0001***	***<0.0001***
*Fasting insulin (µUI/mL)* †	**9.79±5.78**	**13.87±8.8**	**41.04±48.2**	***<0.0001***	***0.029***
*HOMA-IR* †	**1.92±0.96**	**3.57±2.69**	**11.96±13.63**	***<0.0001***	***0.012***
*HbA_1c_ (%)*	**5.02±0.36**	**4.91±0.48**	**5.28±0.29**	***0.002***	***0.002***
*OM BrCa1 (mRNA)*	**6.5±2.5**	**8.8±4.4**	**9.0±5.0**	***0.017***	***0.002***
*OM FASN (mRNA)*	**3.1±1.5**	**1.9±1.4**	**1.7±1.3**	***0.001***	***<0.0001***
*OM ACC (mRNA)*	**3.8±3.3**	**2.0±1.1**	**2.3±1.2**	***0.008***	***0.005***
*OM SREBP-1c (mRNA)*	**2.1±0.9**	**1.7±0.8**	**1.6±0.8**	0.058	***0.022***

*Subpopulation of 33 subjects*	*A. Non-Obese*	*B. Obese*	*C. Obese and T2D*	*P (ANOVA)*	*P (t-Student)*
*n (all women)*	6	13	14		
*Age (years)*	39±8	41±10	47±9	0.170	0.209
*BMI (kg/m^2^)*	**25.8±3.4**	**44.1±4.3**	**43.3±3.1**	***<0.0001***	***<0.0001***
*Fasting glucose (mg/dL)*	**84.7±6.6**	**87.2±12.1**	**170.4±68.8**	***<0.0001***	***0.001***
*SC ACC (ng/mg fat)*	**144.8±119.4**	**19.8±12.6**	**17.2±12.0**	***<0.0001***	0.125
*OM ACC (ng/mg fat)*	101.6±100.4	41.6±34.6	35.4±28.6	0.100	0.297
*Ratio SC P-ACC/ACC*	**0.40±0.16**	**0.69±0.19**	**0.80±0.26**	***0.014***	***0.007***
*Ratio OM P-ACC/ACC*	**0.23±0.13**	**0.50±0.22**	**0.47±0.10**	***0.037***	***0.010***

*Subpopulation of 11 subjects*	*A. Non-Obese*	*B. Obese*	*P (t-Student)*
*n (all women)*	5	6	
*Age (years)*	42±9	47±5	0.227
*BMI (kg/m^2^)*	**26.9±4.2**	**43.6±4.1**	***<0.0001***
*Fasting glucose (mg/dL)*	92.8±19.0	87.7±13.0	0.608
*OM BrCa1 (protein)*	**2.98±0.46**	**4.02±0.52**	***0.007***
*OM P-BrCa1 (protein)*	**3.27±0.55**	**4.89±1.11**	***0.016***
*OM Ratio P-BrCa1/BrCa1*	1.11±0.19	1.22±0.28	0.457

Data are means ±SD for **Non-Obese** (BMI<30 Kg/m^2^), and **Obese** (BMI≥30 Kg/m^2^) subjects with and without type 2 diabetes (**T2D**). **BMI**, Body Mass Index; **HbA_1c_**, glycated hemoglobin; **OM**, omental fat depots; **SC**, subcutaneous fat depots; **BrCa1**, breast cancer protein 1; **P-BrCa1**, phosphorylated BrCa1; **SREBP-1c**, sterol regulatory element-binding protein-1c; **FASN**, fatty acid synthase; **ACC**, acetyl-CoA carboxylase; **P-ACC**, phosphorylated ACC.

† Measures of **fasting insulin**, and the homeostatic model assessment-insulin resistance (**HOMA-IR**) were available in 62 individuals (64.5% of the whole cohort). ***t*** **-Student** was performed for comparisons between non-obese subjects, and merged values from obese subjects with and without T2D. Significant data are shown in **bold**.

Consistent with increased BrCa1 in obesity, increased P-ACC levels were shown in both SC (1.9-fold, p = 0.007; ***Fig. 1b***) and OM (2.1-fold, p = 0.010; ***Fig. 1d***) adipose tissue from obese subjects (***Table 1***), and in SC when compared to OM fat depots (1.7-fold, p = 0.001; ***Fig. S1b***). As expected, both *ACC* gene expression and protein levels, and *FASN* gene expression were decreased in obese subjects when compared to non-obese individuals (***Table 1***). Also *SREBP-1c* gene expression was lower in SC and OM fat from obese subjects with or without T2D (***Table 1***).

In the whole cohort, *BrCa1* mRNA in SC fat was significantly associated with BMI (r = 0.265, p = 0.009), fasting glucose (r = 0.256, p = 0.011), and LDL-cholesterol (r = 0.300, p = 0.008), while OM *BrCa1* gene expression was also associated with BMI (r = 0.343, p = 0.001) and fasting glucose (r = 0.389, p<0.0001), fasting insulin (r = 0.310, p = 0.011), HOMA-IR (r = 0.335, p = 0.007), and LDL-cholesterol (r = 0.300, p = 0.008). Interestingly, fasting glucose contributed independently to 19.6% (p = 0.02) of OM and 15.1% (p = 0.05) of SC *BrCa1* gene expression variance after controlling for the effects of sex, age, and BMI in a multiple linear regression model.

On the other hand, *BrCa1* gene expression in isolated human adipocytes was increased 1.7-fold (p<0.0001; ***Fig. S2***) in mature adipocytes isolated from both SC (n = 12) and OM (n = 12) adipose tissue samples when compared with stromal-vascular cells (SVCs). The higher *BrCa1* gene expression in isolated mature adipocytes was in parallel to the increased *FASN* (∼39-fold, p<0.0001), *ACC* (∼7-fold, p<0.0001), and *SREBP* (∼5-fold, p<0.0001) gene expression levels (***Figure S2***).

### *BrCa1* in cultured adipocytes

**Figure 2:**
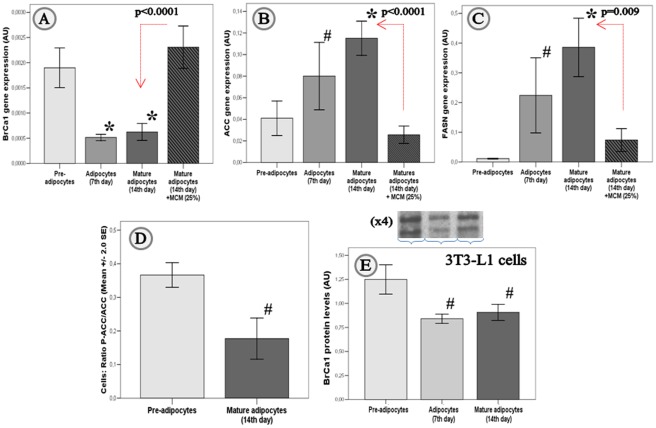
*BrCa1* in cultured adipocytes. *Real Time-PCR:* Mean ±2.0 SE of gene expression levels for *BrCa1* (*Fig. 2a*), *ACC* (*Fig. 2b*), and *FASN* (*Fig. 2c*) during adipogenesis of human fat cells at days 0, 7^th^ and 14^th^ (n = 3/day) after inducing differentiation, and in mature adipocytes after 48 h treatment of macrophage conditioned medium (+MCM; 25%). *ELISA:* Mean ±2.0 SE for phosphorylated ACC normalized *versus* ACC total (ratio P-ACC/ACC) after *in vitro* differentiation (*Fig. 2d*) of human pre-adipocytes to mature adipocytes (n = 3/day). *Western-blot:* Mean ±2.0 SE of protein levels for BrCa1 during differentiation of 3T3-L1 (*Fig. 2e*; n = 4/day). * p<0.0001 and **^#^** p<0.05 for comparisons between levels assessed in adipocytes at 7^th^ and/or 14^th^ day after inducing differentiation *in vitro*, and pre-adipocytes.

*BrCa1* gene expression decreased during *in vitro* differentiation of human pre-adipocytes (−77.4%, p<0.0001, at day 7, and −66.2%, p<0.0001, at day 14; ***Fig. 2a***) in parallel to increased expression levels of SREBP1 (∼5-fold, p = 0.03), *FASN* (∼57-fold, p = 0.04; ***Fig. 2b***), and *ACC* (∼3-fold, p = 0.005; ***Fig. 2c***), and decreased P-ACC/ACC ratio (2.1-fold, p = 0.005; ***Fig. 2d***).

The finding of decreased *BrCa1* expression in mature adipocytes *vs* pre-adipocytes was in contrast with the results for comparisons between mature adipocytes and SVCs isolated from adipose tissue of obese individuals. Thereby we sought to evaluate the immunomodulating effects of inflammatory stimulus such as the macrophage conditioned medium (MCM; 25%) on mature adipocytes. Macrophages have been showed to be at the onset of metabolic diseases by triggering sustained inflammation [34]. THP-1 cells stimulated with lipopolysaccharides (LPS) released to the media high levels of pro-inflammatory cytokines such as IL-8, monocyte chemotactic protein-1, IL-6, and tumor necrosis factor-α, among others [35]. Being this model suitable to study the first potential events leading to the impairment of mature adipocytes behavior, our data suggest that the expression of *BrCa1* could be at the onset of decreased lipogenesis in human adipocytes under inflammatory conditions.

Interestingly, macrophage conditioned medium (MCM; 25%) restored *BrCa1* gene expression in mature adipocytes to the levels assessed for pre-adipocytes (+270%, p<0.0001), whereas *ACC* (−78%, p<0.0001) and *FASN* (−82%, p = 0.009) gene expression was significantly down-regulated (***Fig. 2***). To further substantiate these findings, we confirmed decreased BrCa1 protein (−20.7%, p = 0.010, at day 7, and −18.9%, p = 0.017, at day 14) during differentiation of 3T3-L1 cells (***Fig. 2e***).

## Discussion

Decreased lipogenesis in obese subjects, characterized and defined by increased body fatness, is repeatedly found in the literature (summarized in reference [6]). No consistent mechanistic insights have been reported to explain this contradictory observation. In this respect, sterol regulatory element-binding protein (SREBP)-1c is a well established key transcription factor for the regulation of lipogenic enzymes in hepatocytes but the mechanisms of lipogenic genes regulation in adipocytes remains unclear. Of note, the genetic disruption of SREBP-1 did not cause changes in lipogenic gene expression in adipose tissue [36], and increments in SREBP-1c are not accompanied by transactivation of lipogenic genes in adipocytes, unlike in hepatocytes [37].

In the current study, we evaluated *BrCa1* gene expression and protein levels in human adipose tissue, and in human and murine adipocytes. The principal findings were *i)* the increased expression levels of *BrCa1* in both omental (OM) and subcutaneous (SC) fat depots from obese subjects; *ii)* the higher expression found in SC when compared to OM fat, *iii)* the increased expression of *BrCa1* mRNA in mature adipocytes when compared with stromal-vascular cells (SVCs), and *iv)* the down-regulation of *BrCa1* gene expression and protein during differentiation of both human and murine adipocytes, which can be restored by inflammatory stimuli (i.e. the macrophage LPS-conditioned medium). It can be concluded that the findings of increased BrCa1 in adipose tissue and during adipogenesis were specular to those of lipogenic enzymes such as ACC and FASN.

Although its biological function is not completely understood, BrCa1 seems to have an important role in both cellular differentiation and proliferation [22], [24]. Recent studies have provided structural evidence for direct interactions between BrCa1 and ACC [26]. BrCa1 helps to control fatty acid biosynthesis and lipogenesis in normal cells [27], [29]. Other studies have demonstrated that BrCa1 affects lipid synthesis by preventing ACC dephosphorylation [30]. Our findings are the first to suggest, to our knowledge, a close crosstalk between BrCa1, lipogenesis, adipogenesis, obesity, and obesity-associated insulin resistance.

In the past, variations of *BrCa1* expression were observed during postnatal mammary gland development [21], [38] and Cre-mediated invalidation of this gene affected final differentiation of the gland during gestation [39]. In humans, the up-regulation of *BrCa1* gene expression observed during the first stages of the mammary gland also suggested a role for BrCa1 in the differentiation of the mammary gland [39].

Taking together previous studies and current findings, we propose that long-lasting fat excess tends to reduce lipogenesis in adipose tissue as a process aimed at limiting further expansion of fat mass. Thereby, increased *BrCa1* expression in obesity could be interpreted, as well as the reduced expression for many lipogenic factors [4], [6], as the process through which cells reduce their ability to synthesize additional fatty acids once the storage capacity limit of the adipocytes is attained. The decreased activity of ACC in obesity may be accomplished by increased BrCa1 levels. Accordingly, the phosphorylation degree for ACC was increased in adipose tissue from obese subjects when compared to non-obese individuals, as well as in SC regarding to OM fat depots, and in pre-adipocytes when compared to mature adipocytes. Therefore, decreased capacity for fatty acid biosynthesis in pre-adipocytes and in adipose tissue from obese subjects is confirmed not only through decreased expression of lipogenic enzymes but also by reducing their respective specific activities, as previously suggested [40].

We also sought to assess the impact of adipogenesis on *BrCa1* expression. The expression of FASN and ACC, two well-established adipogenic markers, increased, as expected, during differentiation of pre-adipocytes. Simultaneously, down-regulation of both BrCa1 gene expression and protein levels (in human adipocytes and 3T3-L1 cells) was observed. Since BrCa1 is able to diminish lipogenic activity by interacting with ACC, its down-regulation at the early stages of differentiation and later in mature adipocytes could be necessary in order to enable adipogenesis of human pre-adipocytes.

*BrCa1* gene expression levels were significantly increased in mature adipocytes when compared with SVCs. This again suggests some kind of regulation in adipose tissue from obese subjects with a large and long-lasting fat excess. The increase in *BrCa1* gene expression in mature adipocytes might counteract endogenous lipid synthesis through maintaining ACC phosphorylation and its subsequent inactivation [29]. The decreased expression of lipogenic enzymes and lipid biosynthesis in adipose tissue from obese subjects consistently found in the literature [4], [5], [6], [8] agrees with this observation.

Adipose tissue is important for the production and storage of cholesterol, as reviewed by Miettinen and Tilvis [41]. Moreover, a low capacity of large adipocytes to fill up with more lipids could play a role in dyslipidaemia [42]. It is possible that increased fat cell size in obesity has an impact on lipid metabolism, reducing fatty acid biosynthesis but also decreasing the uptake of circulating lipids leading to increased LDL-cholesterol.

On the other hand, inflammation requires the activation of cellular pathways that reduce deleterious effects in cells. Currently, emerging evidence has indicated that BrCa1 is involved in ROS production and oxidative stress responses [43], which can explain the increased *BrCa1* expression in fat samples from obese subjects. Of note, if the effects of an inflammatory scenario raise *BrCa1* expression in mature adipocytes from obese subjects, is it possible that inflammatory insults can alter the metabolic phenotype of these cells *in vitro*, as previously demonstrated [35], and also *BrCa1* gene expression. Accordingly, we found an increased expression of *BrCa1* in mature adipocytes after the treatment of macrophage LPS-conditioned medium.

Finally, BrCa1 name (Breast Cancer-1) cannot be forgotten. Examination of adipose tissue biology may provide important insight into mechanistic links for the observed association between higher body fat and the risk of several types of cancer, in particular breast cancer [44].

In conclusion, the findings reported here are compatible with the notion that chronic subclinical inflammation of obesity and insulin resistance down-regulates the lipogenic pathway in humans. BrCa1 might be involved in this process during periods of long-lasting energy excess. Otherwise, *BrCa1* down-regulation in pre-adipocytes seems to be necessary in order to allow adipogenesis.

## Methods

### *In vivo* studies

#### Subjects and samples

88 omental (OM) and 96 subcutaneous (SC) adipose tissue samples (51-paired fat samples) were obtained from the lower abdominal wall/inguinal region of both human fat depots during elective surgical procedures (cholecystectomy, surgery of abdominal hernia and gastric by-pass surgery). During surgery, biopsies of adipose tissues were obtained after an overnight fast, washed in chilled 9 g/L NaCl solution, partitioned into pieces, and immediately frozen in liquid nitrogen and stored at −80°C until protein extraction. The surgeon aimed to obtain the samples from similar anatomical locations in all the subjects. These fat samples were provided from a whole cohort of 133 subjects (50 men and 83 women) with a body mass index (BMI) between 20 and 70 kg/m^2^ who were invited to participate at the Endocrinology Service of the *Hospital Universitari de Girona Dr. Josep Trueta* (Girona, Spain), the *Clinica Universitaria de Navarra* (Navarra, Spain), and the *Hospital Carlos Haya de Málaga* (Málaga, Spain). Total and phosphorylated (P-) acetyl-CoA carboxylase (ACC) levels were measured by ELISA in adipose tissue from 33 individuals (6 non-obese and 27 obese subjects). Total BrCa1 protein and phosphorylated (P-) BrCa1 were also analyzed in 11 subjects (6 obese and 5 non-obese) by western-blot.

All subjects were of Caucasian origin and reported that their body weight had been stable for at least three months before the study. They had no systemic disease and all were free of any infections within the previous month before the study. Liver disease and thyroid dysfunction were specifically excluded by biochemical work-up. Other exclusion criteria for those patients included the following: 1) clinically significant hepatic, neurological, or other major systemic disease, including malignancy; 2) history of drug or alcohol abuse, defined as >80 g/day, or serum transaminase activity more than twice the upper normal range limit; 3) elevated serum creatinine concentrations; 4) acute major cardiovascular event in the previous 6 months; 5) acute illnesses and current evidence of chronic inflammatory or infectious diseases; and 6) mental illness rendering the subjects unable to understand the nature, scope, and possible consequences of the study. All subjects gave written informed consent after the purpose of the study was explained to them. The experimental protocol was approved by the Ethics Committee of all participant institutions, including the *Hospital Universitari de Girona Dr. Josep Trueta* (Girona, Spain), the *Clinica Universitaria de Navarra* (Navarra, Spain), and the *Hospital Carlos Haya de Málaga* (Málaga, Spain), respectively, so we certify that all applicable institutional regulations concerning the ethical use of information and samples from human volunteers were followed during this research.

#### Anthropometric and analytical measurements

BMI was calculated as weight (in kilograms) divided by height (in meters) squared. *Deurenberg's* formula was used to estimate body fat composition in those subjects [45]. The serum glucose levels were measured in duplicate by the glucose oxidase method with a *Beckman Glucose Analyzer 2* (*Brea, CA*). The coefficient of variation (CV) was 1.9%. Total serum cholesterol was measured through the reaction of cholesterol esterase/oxidase/peroxidase, using a *BM/Hitachi 747*. Low-density (LDL) and high-density (HDL) cholesterol were quantified after precipitation with polyethylene glycol at room temperature. Total serum triglycerides were measured through the reaction of glycerol-phosphate-oxidase and peroxidase, as previously described [46]. Whole blood hemoglobin levels (*EDTA sample, Coulter Electronics, Hialeah, FL*) were determined by routine laboratory tests.

### Experimental procedures

#### Cell isolation

Approximately 5 g of both subcutaneous (SC) and omental (OM) adipose tissue samples obtained during the surgical procedures carried out in 12 subjects were aseptically isolated and all visible connective tissue was removed. Tissues were finely minced and subjected to a 1 h digestion at 37°C in a shaking water bath. The digestion buffer included 100 mM HEPES (*Sigma Aldrich*) buffer containing 120 mM NaCl, 50 mM KCl, 5 mM D-glucose, 1 mM CaCl2, 1.5% type-V BSA, 2% penicillin/ streptomycin (P/S) and 0.15% collagenase Type I solution (*Sigma Aldrich*). The collagenase Type I solution used to isolate stromal-vascular cells (SVCs) and mature adipocytes (MAs) from fat samples contained approximately 1.5 mg collagenase Type I/mL (*CLS type 1, Worthing Biochemical Corp*). The remaining procedure was similar to the previously described method for isolating SVCs from adipose tissue [47]. Briefly, upon disintegration of the adipose tissue aggregates, digested tissue was centrifuged and two cellular fractions, a pellet of SVCs and a supernatant of MAs, were placed in 20 ml of PBS 2% P/S and passed through sterile nylon mesh filters (autoclaved metal screen, *BD Biosciences*) to isolate digested cells. Finally, both SVCs (n = 12) and MAs (n = 12) filtered fractions were washed and centrifuged for 5 min at 400 *g* before be stored at −80°C. All these subjects were obese women (BMI≥30 kg/m^2^).

#### Cell culture

Commercially available cryo-preserved human subcutaneous pre-adipocytes from one male subject with age ≥40 and BMI≤25 (SP-F-1; *Zen-Bio, Inc.*) were placed on T-75 cell culture flasks and cultured at 37°C and 5% CO_2_ in Dulbecco's modified Eagle's medium (DMEM)/Nutrient Mix F-12 medium (1∶1, v/v) supplemented with Fetal Bovine Serum (FBS) 10%, HEPES 1%, glutamine 1% and Penicillin/Streptomycin at 10 U/mL (all from *GIBCO*, BRL; *Grand Island, NY*). One week later, human SC pre-adipocytes were resuspended and cultured (∼40.000 cells/cm^2^, 3^rd^ passage) in 6-well plates with Pre-adipocyte Medium (PM; *Zen-Bio, Inc.*) composed of DMEM/Nutrient Mix F-12 medium (1∶1, v/v), FBS 10%, HEPES 1%, glutamine 1% and P/S 1% in a humidified 37°C incubator with 5% CO2. Twenty-four hours after plating, cells were checked for complete confluence (day 0) and differentiation was induced using Differentiation Medium (DM; *Zen-Bio, Inc.*), composed of PM with human insulin, dexamethasone, isobutylmethyl-xanthine and PPARγ agonists (Rosiglitazone). After 7 days (day 7), DM was replaced with fresh Adipocyte Medium (AM; *Zen-Bio Inc.*), composed of DMEM/Nutrient Mix F-12 medium (1∶1, v/v), FBS, HEPES, biotin, panthothenate, insulin, dexamethasone, penicillin, streptomycin and amphotericin, according to manufacturers' guidelines. Two weeks after the initiation of differentiation (day 14), cells appeared rounded with large lipid droplets apparent in the cytoplasm. Cells were then considered mature adipocytes (MAs), harvested and stored at −80°C for RNA and protein extraction to study gene expression and protein levels.

Murine 3T3-L1 fibroblasts (CCL 92.1, *American Type Culture Collection*) were grown to confluence at 37°C in 6-well plates in DMEM with no added biotin or pantothenate, containing 10% calf serum in incubators equilibrated with 10% CO2. Two days post-confluence (day 0) differentiation was induced with, isobutylmethyl-xanthine (0.5 mM), dexamethasone (0.25 µM) and Insulin (1 pg/ml) in DMEM containing 10% fetal bovine serum. After 2 days, isobutylmethyl-xanthine and dexamethasone were removed and Insulin was maintained for 2 additional days. On day 4, and thereafter, DMEM (without insulin supplementation) plus 10% FBS was replaced every 2 days. On days 0, 7 and 14 before starting differentiation protocol, four replications of cells were collected separately for total protein extraction. Cell samples were washed in ice-cold PBS followed by homogenization assay using RIPA lysis buffer (*Upstate*) supplemented with a protease inhibitor cocktail (*Sigma Aldrich*) at 4°C for 30 min. Cellular debris were eliminated by centrifugation of the diluted samples at 10,000 g for 10 min (4°C). Protein concentration was then determined using *Lowry* assay.

#### Effects of macrophage-conditioned medium (MCM) on *BrCa1* expression

*In vitro* cultured mature adipocytes (14^th^ day post-differentiation) were further incubated (additional 48 h) with fresh media (control), or with fresh media containing MCM medium (25%), obtained from differentiated human monocyte cell lines (THP-1; *American Type Culture Collection*) treated with medium stimulated with 10 ng/ml LPS (Sigma Chemical) for 24 h, as previously described [35]. After 48 h, mature adipocytes were isolated, harvested and stored at −80°C for RNA extraction.

### Gene expression analyses

RNA was prepared from both adipose tissue and human cellular debris using RNeasy Lipid Tissue Mini Kit (*QIAgen; Gaithersburg, MD*). The integrity of each RNA sample was checked by either agarose gel electrophoresis or with an Agilent Bioanalyzer® (*Agilent Technologies; Palo Alto, CA*). Total RNA was quantified by means of a spectrophotometer (*GeneQuant, GE Health Care; Piscataway, NJ*) or with the Bioanalyzer® and 3 µg of RNA was then reverse transcribed to cDNA using High Capacity cDNA Archive Kit (*Applied Biosystems; Darmstadt, Germany*) according to the manufacturer's protocol.

Gene expression was assessed by real time PCR using the LightCycler® 480 Real-Time PCR System (*Roche Diagnostics; Barcelona, Spain*), using TaqMan® technology suitable for relative gene expression quantification. The reaction was performed following manufacturers' protocol in a final volume of 7 µl. The cycle program consisted of an initial denaturing of 10 min at 95°C then 45 cycles of 15 sec denaturizing phase at 92°C and 1 min annealing and extension phase at 60°C. Replicates and positive and negative controls were included in all the reactions.

The commercially available and pre-validated TaqMan® primer/probe sets used were as follows: breast cancer 1 early onset (*BrCa1; Hs00173233_m1*), sterol regulatory element binding transcription factor 1 (*SREBF1; Hs00231674_m1*), fatty acid synthase (*FASN; Hs00188012_m1*), and acetyl-coenzyme A carboxylase alpha (*ACACA; Hs00167385_m1*). Cyclophilin A (*PPIA; Hs99999904_m1*) and ribosomic 18S (*18S; Hs99999901_s1*) were analyzed and tested as endogenous control in each reaction. The Second Derivative Maximum Method was used for the determination of the crossing points (Cp). A Cp value was obtained for each amplification curve and ΔCp value was first calculated by subtracting the Cp value for each endogenous control from the Cp value for each sample and transcript. Fold changes were then determined by calculating 2^−ΔCp^. Findings normalizing by either PPIA or 18S as endogenous control were correlated (r = 0.65, p<10^−5^; *data not shown*). Gene expression results are shown in all cases in arbitrary units (AU) as expression ratio relative to PPIA according to manufacturers' guidelines.

### Immunoblotting

For protein detection in Western blot, fat tissue and cellular debris were homogenized in radioimmnuno precipitation assay (RIPA) buffer (0.1% SDS, 0.5% sodium deoxycholate, 1% Nonidet P-40, 150 mM NaCl, 50 mM Tris-HCl, pH 8.0), supplemented with protease inhibitors (1 mM phenylmethylsulfonyl fluoride, 2 g/ml aprotinin and 2 g/ml leupeptin). Cellular debris and lipids were eliminated by centrifugation of the solubilized samples at 13,000 rpm for 30 minutes, recovering the soluble fraction below the fat supernatant and avoiding the un-homogenized material at the bottom of the centrifuge tube. Protein concentration was determined by BCA Protein Assay (*Pierce; Rockford, IL, USA*). RIPA protein extracts (ca. 10 µg) were loaded and resolved on 7–14% SDS-PAGE and transferred to Hybond ECL nitrocellulose membranes by conventional procedures. Membranes were stained with 0.15% Ponceau red (*Sigma-Aldrich; St Louis, MO, USA*) to ensure equal loading after transfer and then blocked with 5% (w/v) BSA or dried nonfat milk in TBS buffer with 0.1% Tween 20. The antibodies used for WB analysis revealed in each case single bands at the expected molecular masses. The primary antibodies used were: from *Cell Signaling* rabbit anti-phospho-BrCa1 (Ser1524); from *Santa Cruz Biotechnology* rabbit anti-BrCa1 and goat anti-Beta-Actin; and from *Ambion* mouse anti-GAPDH. Blots were incubated with the appropriate IgG-HRP-conjugated secondary antibody. Immunoreactive bands were visualized with ECL-plus reagent kit (*GE Healthcare*). Blots were exposed for different times; exposures in the linear range of signal were selected for densitometric evaluation. Optical densities of the immunoreactive bands were measured using *Scion Image®* software. Final results are expressed as ratio relative to β-actin for each fat sample or GADPH in 3T3-L1. Statistical comparisons of the densitometry data were carried out using the Student's *t* test, and results were expressed as means ± standard deviation (SD) using SPSS 16.0 (*SPSS Inc., Illinois, USA*). Statistical significance was set at *P*<0.05.

### ELISA

Total acetyl-CoA carboxylase 1 (ACC) and ACC phosphorylated at Serine residue 79 (P-ACC) were measured by enzyme linked immunoassay (ELISA) kit (*Invitrogen Ltd; Paisley, UK*) in lysates of human adipose tissue and cellular debris homogenized by RIPA lysis buffer as previously described (see above). Results from the assay intended for the detection of total ACC independently of phosphorylation status were used for normalization of phosphorylated ACC [pS79]. The analytical sensitivity of these assay were <0.05 ng/mL of ACC total and <0.5 Units/mL of phosphorylated ACC [pS79]. The intra- and inte-rassay CV were, respectively, 3.3% and 6.1% for ACC total, and 4.6% and 5.8% for P-ACC [pS79].

### Statistical analyses

Descriptive results of continuous variables are expressed as mean ±SD. Before statistical analysis, normal distribution and homogeneity of the variances were evaluated using *Levene's* test. One factor ANOVA and *Student*'s *t* test were used to compare variables according groups. Levels of statistical significance were set at *P*<0.05. The relationships between quantitative variables were tested using *Pearson*'s correlation coefficient. The statistical analyses and graphics were performed using the program SPSS (v13.0; *SPSS Inc., Illinois, USA*).

## Supporting Information

Figure S1 **Comparisons between fat depots.** Mean and 95% confidence interval for the mean of gene expression levels for *BrCa1* (*Fig. S1a*), *FASN* (*Fig. S1c*), *ACC* (*Fig. S1d*), and *SREBP-1c* (*Fig. S1d*), and mean ±2.0 SE for phosphorylated ACC normalized *versus* ACC total (ratio P-ACC/ACC) in omental (OM) and subcutaneous (SC) adipose tissue (n = 51 paired samples; *Fig. S1b*). * p<0.05 for comparisons between fat depots.
(TIF)

Figure S2 ***BrCa1*** **in adipocytes and stromal-vascular cells.** Mean and 95% confidence interval for the mean of gene expression levels for *BrCa1* (*Fig. S2a*), *FASN* (*Fig. S2b*), *ACC* (*Fig. S2c*), and *SREBP-1c* (*Fig. S2d*), in stromal-vascular cells (SVCs) and mature adipocytes (MAs) isolated from adipose tissue biopsies (n = 12 paired samples). * p<0.0001 for comparisons between *ex vivo* isolated cells.
(TIF)
