# Supplementary figures and images for: Breast Cancer 1 (BrCa1) May Be behind Decreased Lipogenesis in Adipose Tissue from Obese Subjects
Source: PLoS One. 2012 May 30;7(5):e33233. doi: 10.1371/journal.pone.0033233 (PMC3364252; doi:10.1371/journal.pone.0033233)

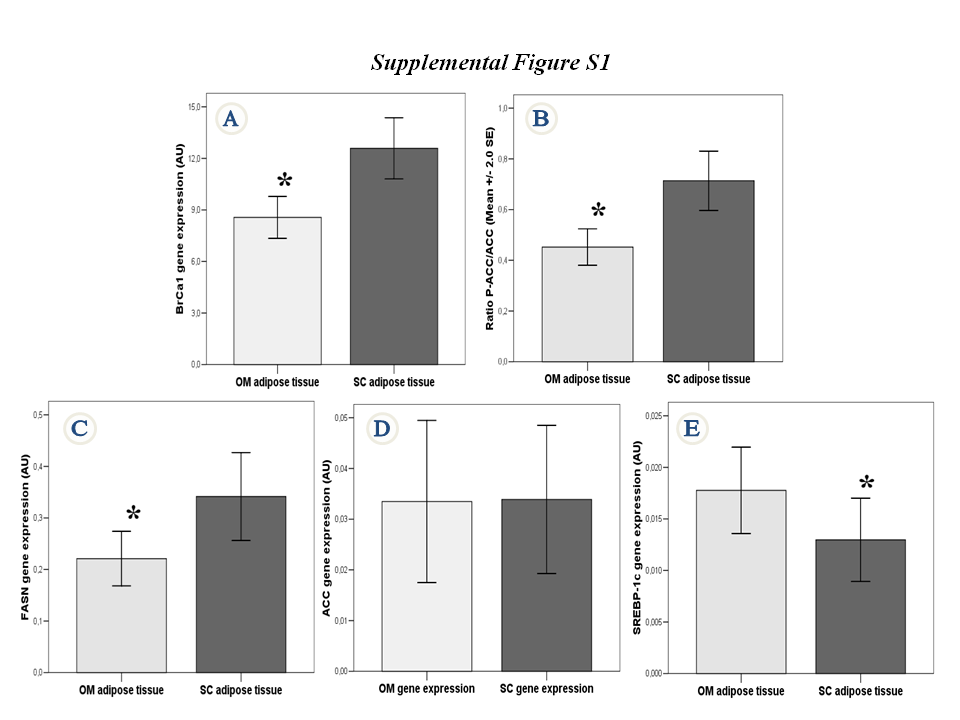

Supplement: Figure S1 — Comparisons between fat depots. Mean and 95% confidence interval for the mean of gene expression levels for BrCa1 (Fig. S1a), FASN (Fig. S1c), ACC (Fig. S1d), and SREBP-1c (Fig. S1d), and mean ±2.0 SE for phosphorylated ACC normalized versus ACC total (ratio P-ACC/ACC) in omental (OM) and subcutaneous (SC) adipose tissue (n = 51 paired samples; Fig. S1b). * p<0.05 for comparisons between fat depots. (TIF) [file pone.0033233.s001.tif]

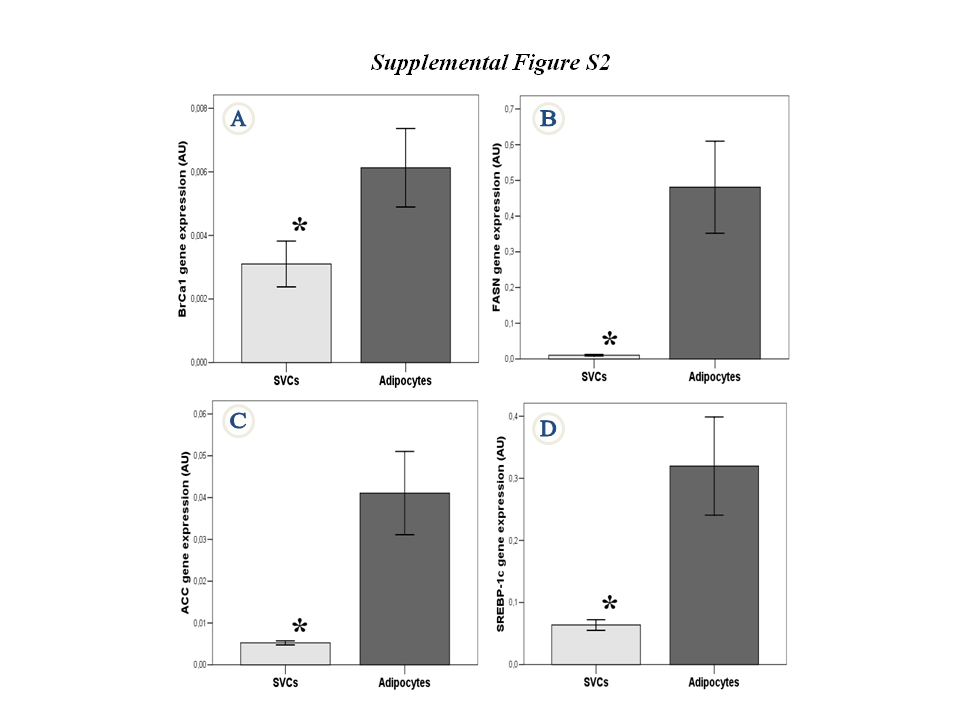

Supplement: Figure S2 — BrCa1 in adipocytes and stromal-vascular cells. Mean and 95% confidence interval for the mean of gene expression levels for BrCa1 (Fig. S2a), FASN (Fig. S2b), ACC (Fig. S2c), and SREBP-1c (Fig. S2d), in stromal-vascular cells (SVCs) and mature adipocytes (MAs) isolated from adipose tissue biopsies (n = 12 paired samples). * p<0.0001 for comparisons between ex vivo isolated cells. (TIF) [file pone.0033233.s002.tif]
